# Prednisolone Attenuates Improvement of Cardiac and Skeletal Contractile Function and Histopathology by Lisinopril and Spironolactone in the *mdx* Mouse Model of Duchenne Muscular Dystrophy

**DOI:** 10.1371/journal.pone.0088360

**Published:** 2014-02-13

**Authors:** Paul M. L. Janssen, Jason D. Murray, Kevin E. Schill, Neha Rastogi, Eric J. Schultz, Tam Tran, Subha V. Raman, Jill A. Rafael-Fortney

**Affiliations:** 1 Department of Physiology and Cell Biology, The Ohio State University, Columbus, Ohio, United States of America; 2 Department of Molecular and Cellular Biochemistry, The Ohio State University, Columbus, Ohio, United States of America; 3 Division of Cardiovascular Medicine, The Ohio State University, Columbus, Ohio, United States of America; 4 Dorothy M. Davis Heart & Lung Institute, The Ohio State University, Columbus, Ohio, United States of America; Stem Cell Research Institute, Belgium

## Abstract

Duchenne muscular dystrophy (DMD) is an inherited disease that causes striated muscle weakness. Recently, we showed therapeutic effects of the combination of lisinopril (L), an angiotensin converting enzyme (ACE) inhibitor, and spironolactone (S), an aldosterone antagonist, in mice lacking dystrophin and haploinsufficient for utrophin (*utrn^+/−^;mdx,* het mice); both cardiac and skeletal muscle function and histology were improved when these mice were treated early with LS. It was unknown to what extent LS treatment is effective in the most commonly used DMD murine model, the *mdx* mouse. In addition, current standard-of-care treatment for DMD is limited to corticosteroids. Therefore, potentially useful alternative or additive drugs need to be both compared directly to corticosteroids and tested in presence of corticosteroids. We evaluated the effectiveness of this LS combination in the *mdx* mouse model both compared with corticosteroid treatment (prednisolone, P) or in combination (LSP). We tested the additional combinatorial treatment containing the angiotensin II receptor blocker losartan (T), which is widely used to halt and treat the developing cardiac dysfunction in DMD patients as an alternative to an ACE inhibitor. Peak myocardial strain rate, assessed by magnetic resonance imaging, showed a negative impact of P, whereas in both diaphragm and extensor digitorum longus (EDL) muscle contractile function was not significantly impaired by P. Histologically, P generally increased cardiac damage, estimated by percentage area infiltrated by IgG as well as by collagen staining. In general, groups that only differed in the presence or absence of P (i.e. *mdx* vs. P, LS vs. LSP, and TS vs. TSP) demonstrated a significant detrimental impact of P on many assessed parameters, with the most profound impact on cardiac pathology.

## Introduction

Duchenne muscular dystrophy (DMD) is an inherited disease that causes striated muscle weakness, for which a cure is currently not available [1]. Pharmacological drug treatment for this disease is limited almost exclusively to corticosteroids, which result in prolonged ambulation in patients for up to 2 years and possibly a delay in respiratory function decline [2], [3], [4], [5], [6], [7]. However, corticosteroid use is known to have adverse side-effects, such as behavioral changes, immune suppression, hypertension, glucose intolerance, bone demineralization, cataracts, myoglobinuria, short stature and delayed puberty [2], which can limit length-of-use and effectiveness. Since the corticosteroid prednisone is currently the standard-of-care for young DMD boys, development of other pharmacological therapies would need to be compared to steroids, as well as tested for impact in presence of steroids, since a placebo-controlled clinical study may not be feasible in ambulatory patients where outcome measures are most easily quantifiable [8], [9], [10].

Cardiac dysfunction in DMD patients is often only considered a secondary focus to skeletal muscle dysfunction. Cardiac dysfunction, however, is a critical component of the DMD disease progression [11], [12] that can be detected early in the disease process [13] before changes in ejection fraction. The adverse impacts of cardiac dysfunction are partially masked by the low physical activity (e.g. that demand a low cardiac output) of DMD patients. Therefore, it is important to incorporate a cardiac function assessment in any study on DMD patients, even if a symptomatic phenotype of cardiac dysfunction is not yet present [14].

Recently, in the search for novel drug-treatment strategies for DMD, we showed efficacy of the combination of lisinopril (L), an angiotensin converting enzyme (ACE) inhibitor, and spironolactone (S), an aldosterone antagonist in mice lacking dystrophin, and heterozygous for utrophin (*utrn^+/−^;mdx,* het mice) [15]. Skeletal and cardiac dysfunction is readily observed at 20 weeks-of-age in this dystrophic model when left untreated. When treated early in the disease process with lisinopril plus spironolactone, we found these het mice had significantly increased cardiac contractile function, and double the diaphragm and skeletal muscle specific contractile force compared to untreated het mice. Histopathological evaluation also showed a significant reduction of ongoing cardiac and skeletal muscle damage compared to untreated mice [15]. We first tested lisinopril/spironolactone in the het mouse model because it shows quantitatively more skeletal muscle fibrosis [16] and cardiac muscle damage than age-matched *mdx* littermates, and therefore provides a larger window to detect therapeutic efficacy. However, since *mdx* mice are the genotypic model of DMD, it is important to validate at least a trend towards therapeutic effects of these drugs in this less affected mouse model, even though in 20-week old *mdx* mice cardiac muscle may be only minimally affected. In order to further evaluate the potential efficacy of this drug treatment regimen for patients with DMD, the combination of lisinopril/spironolactone (LS) also needs to be tested in presence of standard-of-care drug that is typically used in this patient population.

In the present study, we thus sought to evaluate the effectiveness of this lisinopril/spironolactone combination 1) in the *mdx* mouse, and 2) versus steroid treatment alone (prednisolone, P), or in combination with steroid treatment (LSP). In addition, the angiotensin II receptor blocker losartan (T) is widely used to halt and treat the developing cardiac dysfunction in DMD patients as an alternative to an ACE inhibitor, typically lisinopril. Therefore, we added a group in which we evaluated the outcome of treatment with losartan/spironolactone (TS), as well as the same combination in presence of prednisolone (TSP). All experiments were performed in the most commonly used mouse model of muscular dystrophy, the *mdx* mouse [17], [18], with treatments initiated at 4 weeks-of-age, and physiological and histological end-point assessments evaluated at 20 weeks-of-age.

## Materials and Methods

### Ethics Statement

All experiments were approved by the Institutional Animal Care and Use Committee of The Ohio State University and are in compliance with the laws of The United States of America and conform to the Guide for the Care and Use of Laboratory Animals published by the United States National Institutes of Health.

### Treatment of *mdx* Mice

*Mdx* mice from each litter were given numbered ear tags at 3 weeks-of-age. At weaning (4 weeks-of-age), each litter of mice was separated into cages containing either 2 males or 2–3 female mice. Each cage from a litter was started on a different treatment strategy to ensure randomization and so that analysis of each treatment group was interspersed over the time-period of analysis. Equal numbers of males and females were started on each treatment. 2 undergraduate students prepared all of the drug water bottles and were responsible for changing the water bottles for each cage every Monday morning, Wednesday mid-day, and Friday late-afternoon. These 2 students helped to cut histological sections, but did not participate in performing or analyzing any of the function or histological assays. Personnel who performed functional and histological assays were not involved in treating the animals and were blinded to the treatment groups. No more than 5–8 animals were started on treatment within the period of 1 week, since the comprehensive functional analysis could only be feasibly carried out on a maximum of 5–8 animals per week. All animals were analyzed at 20 weeks-of-age.

In total, 7 groups of mice were included in the study; untreated C57BL/10 wild-type mice (C, n = 10), untreated *mdx* mice (U, n = 12), *mdx* mice treated with prednisolone, the active metabolite of prednisone (P, n = 10), *mdx* mice treated with both lisinopril and spironolactone (LS, n = 10), *mdx* mice treated with lisinopril, spironolactone, and prednisolone (LSP, n = 10), *mdx* mice treated with both losartan and spironolactone (TS, n = 11), and *mdx* mice treated with losartan, spironolactone, and prednisolone (TSP, n = 10). Mice were given water bottles with the various drug combinations or without drugs, and were treated for a period of 16 weeks, starting at 4 weeks of age, which in *mdx* mice is before the onset of significant cardiac and skeletal muscle damage/dysfunction. This timing was found to show efficacy for the combination of lisinopril+spironolactone in *mdx* mice also haploinsufficient for the utrophin gene (*utrn^+/−^;mdx*) [15]. The total number of the mice initially included in the study was n = 73. This number was based on previous group sizes, and was logistically restrained by the comprehensive post-treatment analysis. Several factors led to non-equal group numbers; 1) a few mice died during the course of the study, 2) The success rate on *in vitro* muscle physiology is ∼85%, technical issues such as suture-rupture, or equipment failure cause a few experiments to not yield usable data, 3) All mice are housed 2–3 per cage and therefore occasionally an extra mouse was included in a group to avoid confounding effects due to solitary housing of animals. Combined, these factors resulted in a slightly uneven number of mice per group.

Water bottles contained the following concentrations of drugs in the above-described combinations: 66 mg/L lisinopril (“L”; Sigma L 2777); 125 mg/L spironolactone (“S”; Sigma S3378); 600 mg/L losartan (“T”; LKT Laboratories L5873); and 10 mg/L prednisolone (“P”; Sigma P6004). These concentrated equated to approximate dosages of each drug, based on average water consumption, as follows: 10 mg/kgxday lisinopril; 18.75 mg/kgxday spironolactone; 1.5 mg/kgxday prednisolone; 90 mg/kgxday losartan. All dosages were based on allometric scaling of clinical dosages to use in mice (based on the FDA: Guidance for Industry; Estimating the Maximum Safe Starting Dose in Initial Clinical Trials for Therapeutics in Adult Healthy Volunteer) and were dosages that were previously shown to be efficacious in either other *mdx* mouse studies (prednisolone and losartan) or in other mouse cardiomyopathy models (lisinopril and spironolactone) [15].

### *In vivo* Cardiac Analysis

One day before the animals reached 20 weeks-of-age, the animals were anesthetized (isoflurane), and magnetic resonance imaging was performed on a 11.7 Tesla 30 mm bore system (Bruker Biospin, Ettlingen, Germany) with electrocardiographic (ECG) leads while under body temperature control (37°C), as previously described [15]. Myocardial strain and strain rate were computed using vector-based tracking software (Vector Velocity Imaging, Siemens, Mountain View, CA). The next day, prior to sacrifice, body weight of the animals was recorded and ECG’s were recorded in unanesthetized, unrestrained mice using the ECGenie system. From the ECG signals, RR-interval, QT-interval, and heart-rate variability were assessed.

### *In vitro* Diaphragm and Extensor Digitorum Longus (EDL) Contraction Analysis

Isolated strips of diaphragm muscle (n = 1–2/mouse) were assessed on their contractile strength, at different frequencies. At the 2 highest frequencies used, 150 and 180 Hz, a smooth tetanic force was obtained in all muscles. Diaphragm experiments were performed at 37°C to closely mimic physiologically relevant temperature [19]. Specific forces were calculated by dividing the absolute force by the cross-sectional area of the muscle, as described in detail previously [19]. If 2 strips were assessed from the same mouse, values were averaged and treated as n = 1 in the statistical analysis.

Contractile strength of EDL muscle was performed as previously described [20], [21]. Briefly, twitch contractions were used to stretch the muscle to optimal length, and a tetanus was induced by a 250 ms duration stimulation train (150 Hz at 30°C). Thereafter, a series of 10 eccentric contractions were done by application of 450 ms tetani in which during the last 200 ms the muscle was stretched by 3%, whereafter stimulation was halted and the muscle returned to baseline optimal length. Forces were recorded as absolute force, and specific force was calculated by dividing absolute force by cross-sectional area, as described previously. If both EDL muscles were assessed from the same mouse, these two values were averaged and treated as n = 1 in the statistical analysis. EDL mass was assessed after blotting the muscle in between two Kimwipes beneath a 10 g weight for 10 seconds.

### Histopathological Analysis

Both the hearts and quadriceps muscles of the mice were embedded in optimal-cutting-temperature medium and frozen in liquid-nitrogen-cooled isopentane. EDL and diaphragm muscle were not analyzed because the strain caused by functional tests prevents high-quality or complete muscle analysis, whereas only investigating part of the muscle may misrepresent a histological phenotype due to often-occurring localization of damage that may be missed. From these heart and quadriceps specimens, eight micrometer-thick transverse sections were cut. Sections from each muscle from all mice were stained with hematoxylin and eosin by standard methods to assess overall histopathology. Additional sections of both heart and quadriceps from all tissues harvested from all mice collected at 20 weeks-of-age were stained for intracellular immunoglobulin G (IgG) using an Alexa 488 -conjugated goat-anti-mouse IgG antibody (Invitrogen Molecular Probes A11029) (1∶100) and co-stained with anti-Collagen1 antibody (Abcam 292) at 1∶150 and a AlexaFluor 555-conjugated goat-anti-rabbit IgG secondary antibody (Invitrogen Molecular Probes A21429) (1∶200).

Immunostained sections were photographed on a Nikon Eclipse 800 Epifluorescence microscope through a 4X objective using a SPOT Digital Camera and software. The percentage of damaged cardiac and quadriceps muscle tissue was assessed on IgG stained sections using Photoshop CS5.5 (Adobe). Briefly, images were first composited using the automated tools in the program. The paint-bucket tool was used to manually highlight the damaged regions, and the remainder of the section darkened to subtract the normal localization of IgG in capillaries. The number of pixels with non-zero luminosity was used to calculate the percentage of damaged muscle in each composite image. Longitudinal sections of quadriceps were excluded from IgG analysis.

The average percentage of fibers with centrally located nuclei per group was determined by counting 500 contiguous fibers in cross-sections of quadriceps muscles from 3–5 of the mice per group which had IgG staining percentages near the median for each group. Sections that did not have perfect cross-sections or had ice crystal damage were excluded so as not to confound the data analysis and account for the groups that have less than 5 sections counted. Differences between the groups was determined by one-way ANOVA followed by Bonferroni.

### Data Analysis and Statistics

During the study, all data was collected in a blinded fashion, i.e. the experimenter did not know the genotype nor treatment of the mouse. Data were unblinded and grouped only after completion of all analyses. With consult from statisticians, the overall data set was analyzed using ANOVA, with either Tukey, Bonferroni, or Dunnett post-hoc tests, where applicable. A P-value <0.05 was taken as significance level.

## Results

From the initial 73 mice at the start of the study, 8 mice died over the course of the 16-week treatment period. Although the exact cause of death could not be determined, it was noteworthy that none of the C57BL/10 control mice, nor any of the TS-, LS- or P-treated mice died; 1 untreated *mdx* mouse died, and 4 LSP and 2 TSP mice died during the 16-week protocol. 1 female untreated *mdx* mouse died at 4.5 weeks-of-age. 2 extra *mdx* females were added to the study to ensure enough untreated data at the time of analysis. 2 LSP females and 2 LSP males died (at 6, 12, 9, and 6 weeks-of-age, respectively) leaving 6 mice remaining at the time of analysis. 1 TSP female died at 6.5 weeks-of-age, and 1 TSP male died at 19 weeks-of-age leaving 9 mice remaining at the time of analysis.

**Figure 1:**
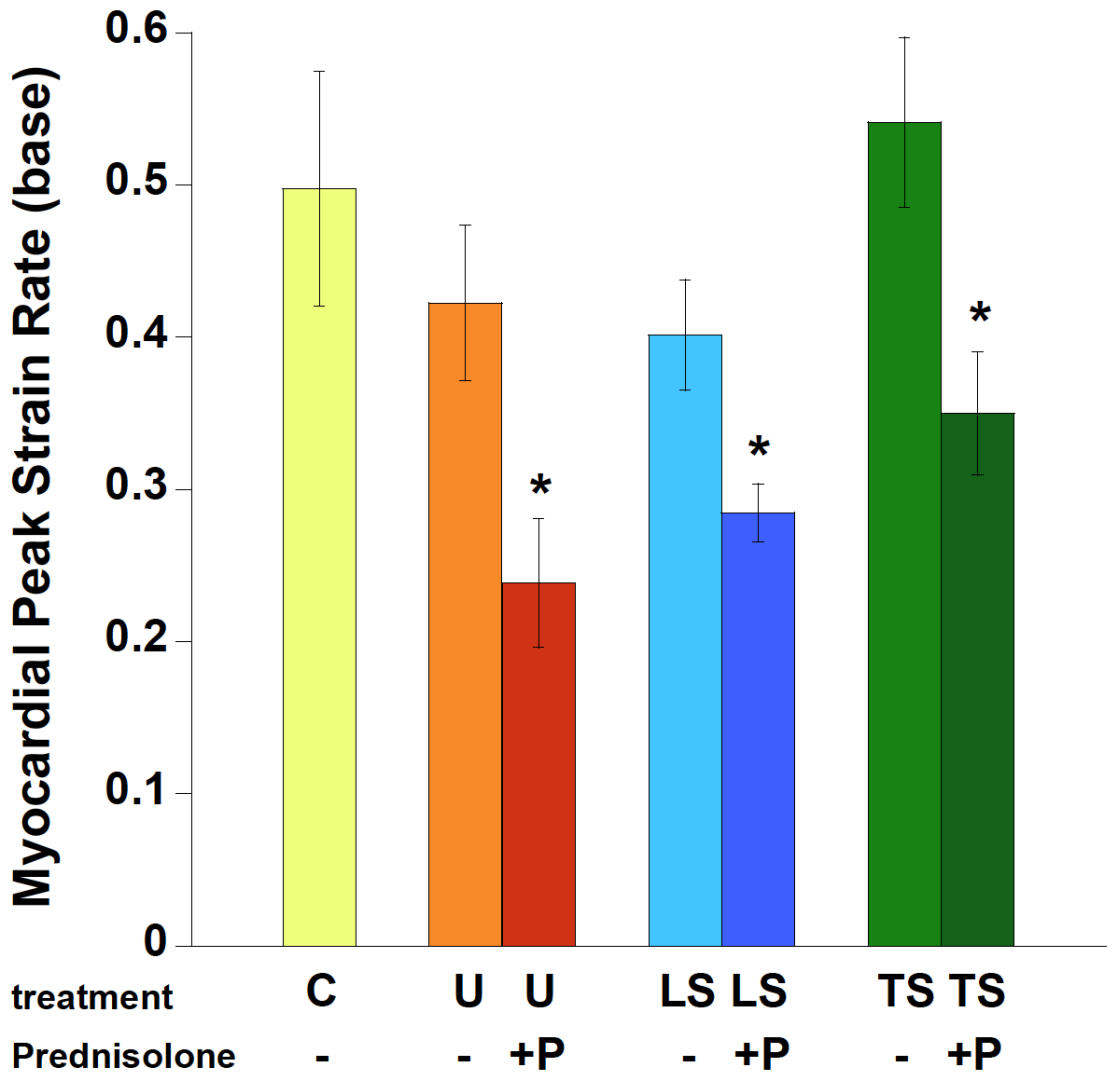
Cine Cardiac Magnetic Resonance Imaging was performed on a subset of mice. Peak circumferential strain rate, assessed at the base of the left ventricle, was negatively impacted when Prednisolone was added to the treatment regimen. C: C57BL/10, n = 4; U: untreated *mdx* mice, n = 5, P: Prednisolone treated *mdx* mice, n = 6, LS: Lisinopril-Spironolactone treated *mdx* mice, n = 5; LSP, Lisinopril-Spironolactone-Prednisolone treated *mdx* mice n = 5; TS: Losartan-Spironolactone treated *mdx* mice, n = 5; TSP: Losartan-Spironolactone-Prednisolone treated *mdx* mice, n = 7. * indicates a significantly lower strain rate compared to the equivalent primary treatment without Prednisolone, P<0.05.

Cardiovascular magnetic resonance imaging was performed on 4–6 mice of each treatment group. Peak systolic strain rate, assessed at the base of the left ventricle, is given in Figure 1. In *mdx* mice, at 20 weeks of age, there is no significant decay yet in this parameter. Interestingly, prednisolone significantly worsened this strain rate, reaching significance when compared to both the untreated *mdx* group, the C57BL/10 group, and compared to the TSP group (ANOVA). When directly comparing groups containing prednisolone with identical groups in absence of prednisolone (i.e. U vs. P, LS vs. LSP, and TS vs. TSP), prednisolone significantly worsened strain rate in each comparison (unpaired t-test, P<0.05).

Next, ECG’s were assessed in all groups. In fully conscious and unrestrained mice, none of the assessed parameters were different between any of the groups. A summary of the heart rate, heart-rate variability, and QT-duration is given in Table 1.

**Table 1:** ECG parameters.

Treatment Group	n	HR	HRV	QT
(Abbreviation)		(bpm)	(bpm)	(ms)
C57BL/10 (C)	8	616±33	98±14	50.2±3.5
*mdx* (U)	7	582±38	67±26	53.5±3.4
*mdx* (P)	9	606±29	46±5	49.1±2.7
*mdx*+LS (LS)	10	631±21	56±7	49.4±1.9
*mdx*+LS+P (LSP)	5	594±13	57±7	50.3±1.8
*mdx*+TS (TS)	11	582±28	73±27	51.8±2.7
*mdx*+TS+P (TSP)	8	524±23	66±5	59.1±2.9
ANOVA		P = 0.19	P = 0.50	P = 0.21

P: prednisolone; LS: Lisinopril/spironolactone; TS: Losartan/spironolactone.

**Figure 2:**
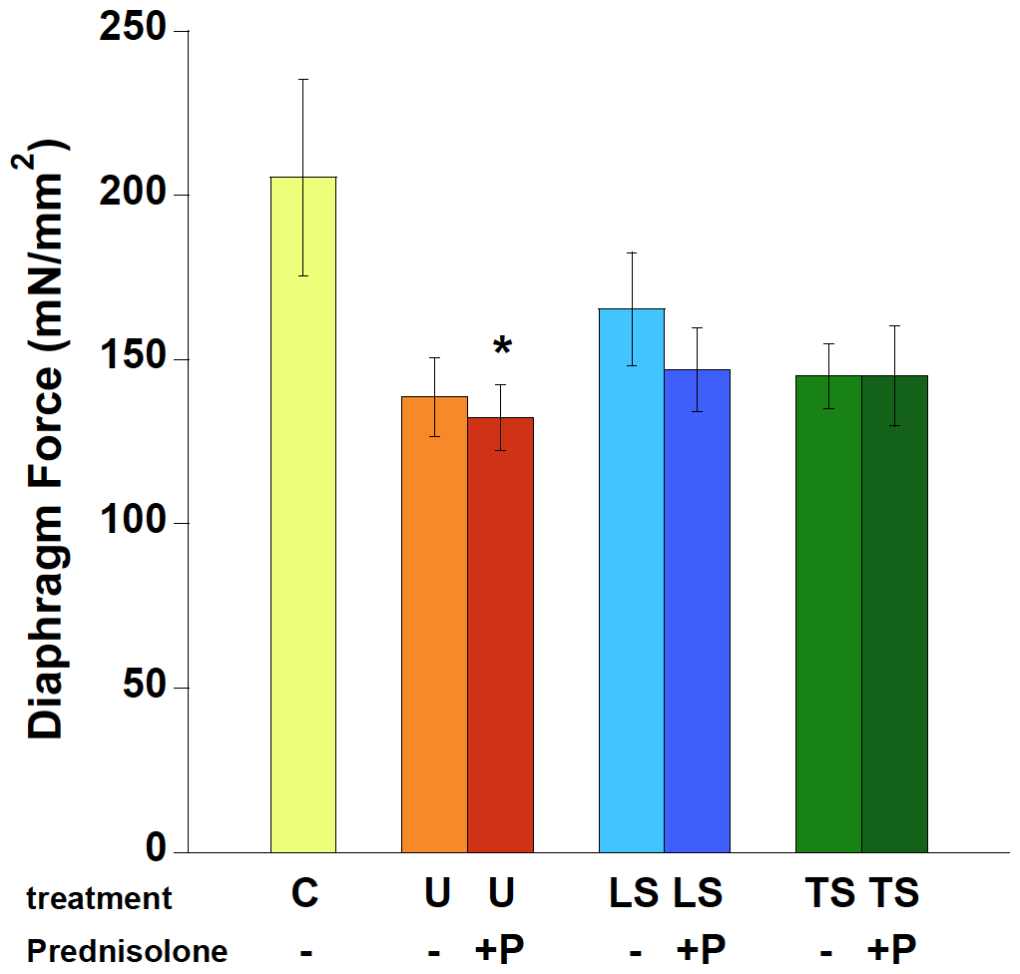
Diaphragm contractile function was assessed in isolated strips, at optimal length, at 37°Celsius. C: C57BL/10, n = 9; U: untreated *mdx* mice, n = 7, P: Prednisolone treated *mdx* mice, n = 10, LS: Lisinopril-Spironolactone treated *mdx* mice, n = 9; LSP, Lisinopril-Spironolactone-Prednisolone treated *mdx* mice n = 6; TS: Losartan-Spironolactone treated *mdx* mice, n = 11; TSP: Losartan-Spironolactone-Prednisolone treated *mdx* mice, n = 8. * ANOVA indicated the only significant difference was between wild-type mice and Prednisolone-treated *mdx* mice (P<0.05). Difference between wild-type and untreated *mdx* mice was very similar but not quite significant (P = 0.08).

Maximal *in vitro* contractile force in diaphragm muscle (Figure 2) is typically depressed by ∼40–50% in *mdx* mice compared to C57BL/10 control mice [19], [22]. Although we found a difference between the means of C and untreated *mdx* (U) mice of 32%, due to the relatively large variance this difference was not quite significant in these particular groups of mice (P = 0.12). ANOVA did however indicate that an overall significance of treatment was detected, and post-hoc analysis revealed a significant difference between C and P mice (P<0.05).

**Figure 3:**
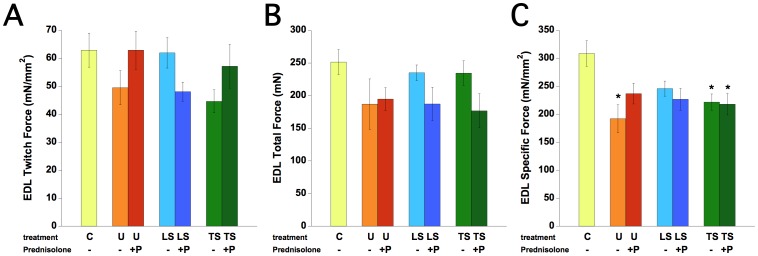
EDL contractile function was assessed in isolated whole EDL muscle, at optimal length, at 30°Celsius. A: Twitch force: Twitch force at optimal length was not significantly different between groups. B: Total force: tetanic force at optimal length was not significantly different between groups. C: Specific force: force normalized to the muscle’s cross-sectional area revealed C57BL/10 mice have significantly higher forces than untreated *mdx* mice, TS, and TSP groups, *P<0.05. C: C57BL/10, n = 8; U: untreated *mdx* mice, n = 6, P: Prednisolone treated *mdx* mice, n = 9, LS: Lisinopril-Spironolactone treated *mdx* mice, n = 10; LSP, Lisinopril-Spironolactone-Prednisolone treated *mdx* mice n = 6; TS: Losartan-Spironolactone treated *mdx* mice, n = 11; TSP: Losartan-Spironolactone-Prednisolone treated *mdx* mice, n = 8.

In EDL muscles, *in vitro* twitch contractions at optimal length were not significantly impacted by treatment, with group averages ranging between 44.6 mN/mm^2^ (TS) to 63.0 mN/mm^2^ (C), shown in Figure 3A. Maximal absolute tetanic contractions were also not significantly impact by treatment (Figure 3B), and group averages ranged from 177 mN (TSP) to 252 mN (C). Specific force (total force divided by muscle cross-sectional area, Figure 3C) was significantly (P<0.05 by ANOVA) impacted by treatment; the control group (C) was significantly (P<0.05) stronger (309±23 mN/mm^2^) than the untreated *mdx* mice (U, 192±25 mN/mm^2^), as well as compared to TS (222±15 mN/mm^2^) and TSP (218±19 mN/mm^2^) treated mice. The other treatment groups (P, LS, and LSP) fell in between U and C, and were not statistically different.

**Figure 4:**
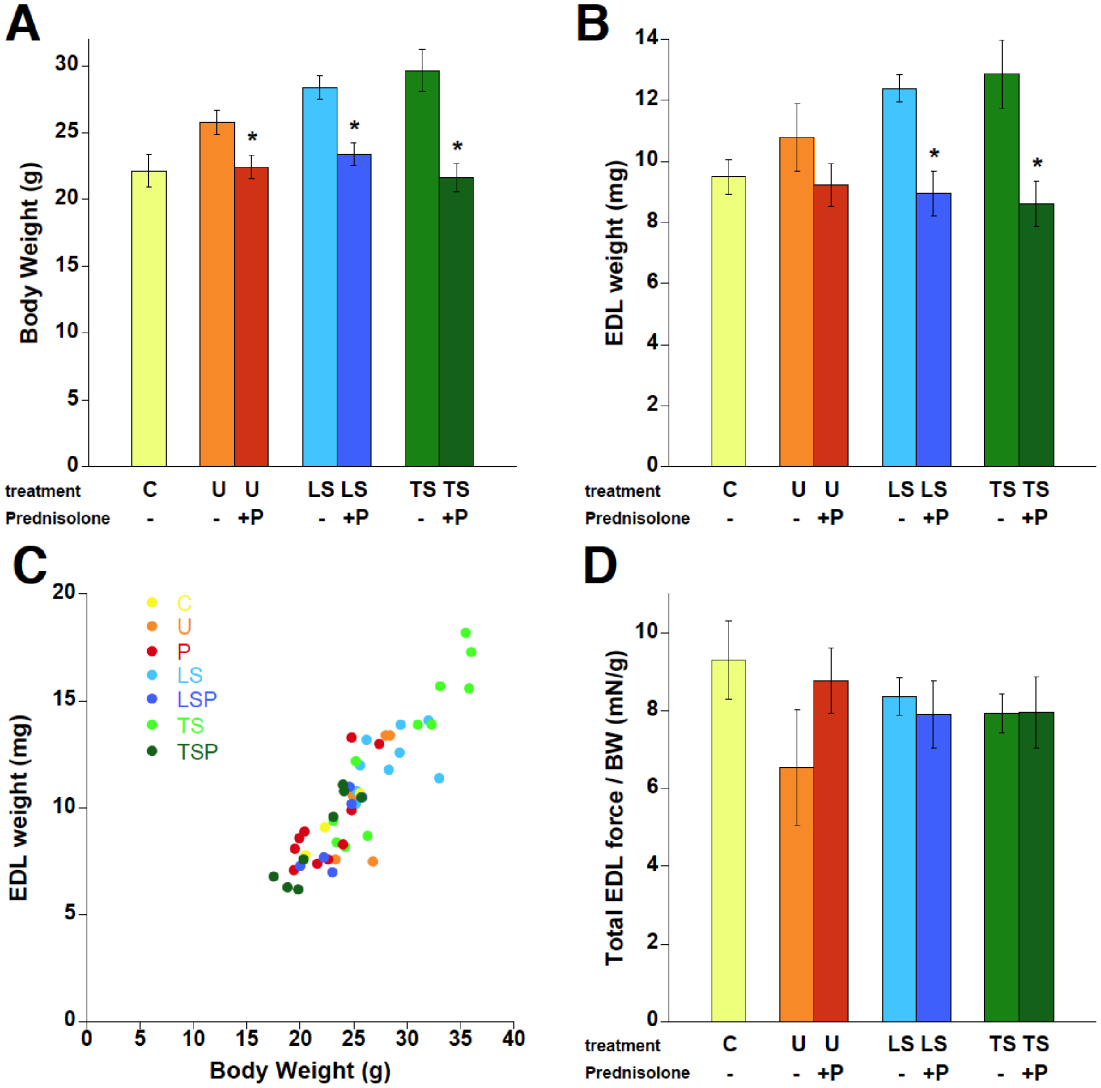
Body weight and EDL weight analysis. A: Body weight was significantly reduced in presence of Prednisolone. B: EDL weight showed a similar pattern to body weight. C: Body weight and EDL weight show a close correlation. D: Total EDL force/BW shows a similar ratio in all groups, with a non-significant (P = 0.09) trend to be lower in untreated *mdx* mice. C57BL/10, n = 4–8; U: untreated *mdx* mice, n = 6, P: Prednisolone treated *mdx* mice, n = 10, LS: Lisinopril-Spironolactone treated *mdx* mice, n = 10; LSP, Lisinopril-Spironolactone-Prednisolone treated *mdx* mice n = 6; TS: Losartan-Spironolactone treated *mdx* mice, n = 11; TSP: Losartan-Spironolactone-Prednisolone treated *mdx* mice, n = 8. * indicates a significantly lower value compared to the equivalent primary treatment without Prednisolone, P<0.05.

Interestingly, body weight (BW) and muscle weight assessment revealed significant differences between various groups (Figure 4A). A direct comparison between *mdx* and C57BL/10 mice revealed a significantly higher BW in *mdx* mice (P<0.05), but the most obvious impact was the increased weight of the LS and TS groups versus both the C and P groups, and the impact of P to decrease weight in all three direct-comparison groups (i.e. U vs. P, LS vs. LSP, and TS vs. TSP). EDL weight showed a similar pattern as body weight (Figure 4B). As a result, when corrected for body weight (i.e. EDL weight/BW), ANOVA indicated no differences in EDL weight by treatment (P = 0.46), and a direct per-mouse comparison of BW and EDL weight revealed a close correlation (Figure 4C). Physiologically, the total force generated by a muscle relative to the body mass needed to be moved is a critical factor for overall organism function and well-being. Thus, as an indicator of whole body physiology, we examined EDL total force divided by body weight. This ratio (highest in C, lowest in U) did not reveal any significant differences between controls and/or treatment groups (Figure 4D, ANOVA, P = 0.61).

**Figure 5:**
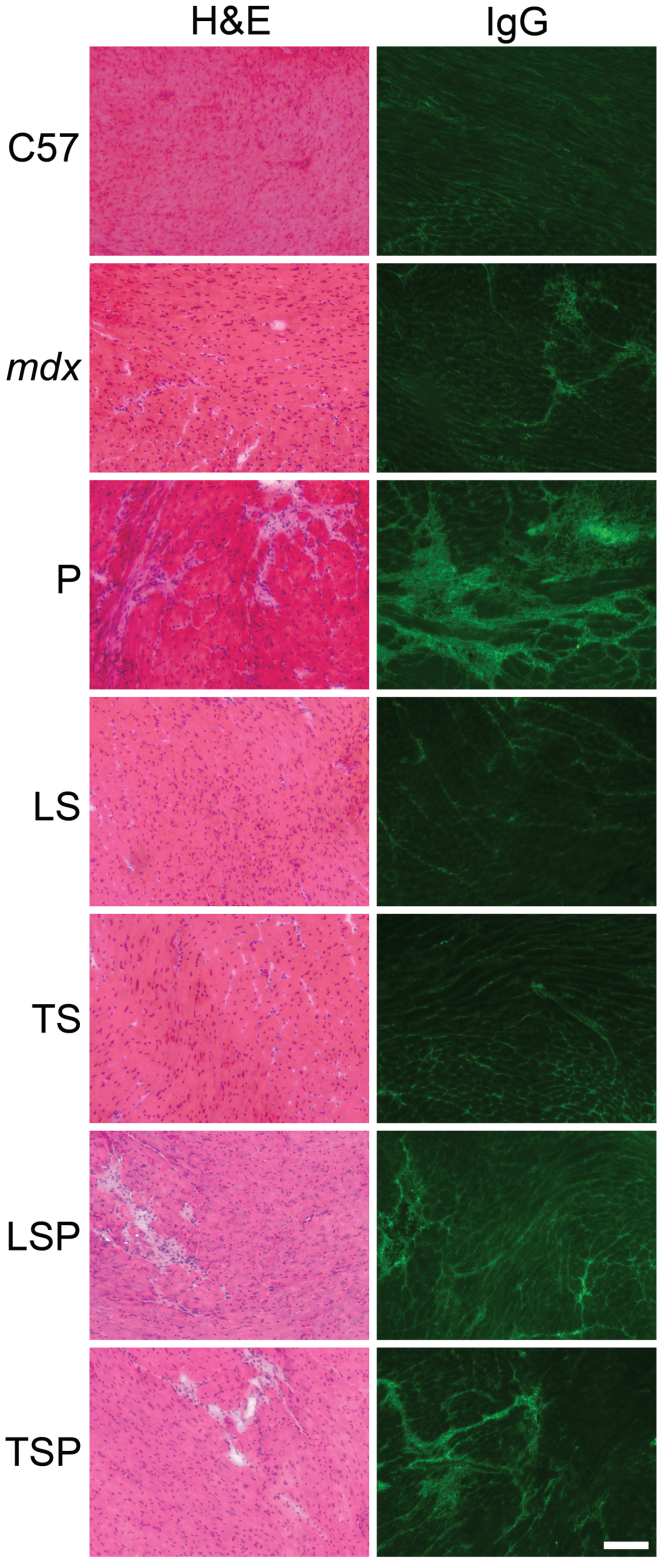
Hematoxylin and eosin (H&E) and IgG stained representative heart sections. Cardiac damage in 20 week-old *mdx* mice is very mild and slightly attenuated by LS treatment. However, P treatment augments the damage present in *mdx* hearts as observed by both IgG stained myocytes as well as regions where cardiac muscle has been replaced by fibrotic scars as observed in H&E stained sections. C: C57BL/10; U: untreated *mdx* mice, P: Prednisolone treated *mdx* mice, LS: Lisinopril-Spironolactone treated *mdx* mice; LSP, Lisinopril-Spironolactone-Prednisolone treated *mdx* mice; TS: Losartan-Spironolactone treated *mdx* mice; TSP: Losartan-Spironolactone-Prednisolone treated *mdx* mice. Bar = 100 µm.

**Figure 6:**
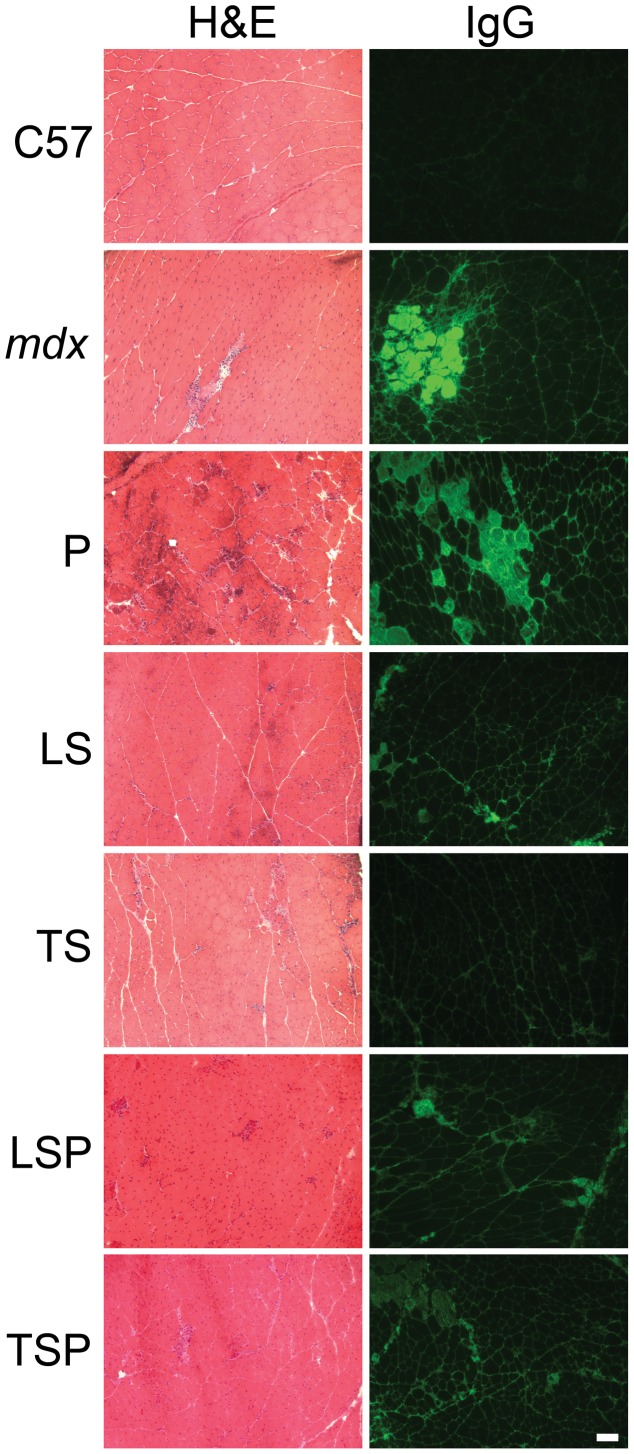
Hematoxylin & eosin (H&E) and IgG stained representative quadriceps sections from each treatment and control group of mice. Small patches of damaged myofibers, representing ongoing skeletal muscle damage at 20 weeks-of-age are present in *mdx* quadriceps muscles (bright green IgG staining), but are completely absent from 20 week-old wild-type mice (C57BL/10). LS and TS treatment reduces the amount of ongoing damage, but P treatment results in larger and more prevalent patches of ongoing skeletal muscle damage in *mdx* quadriceps muscles. The same pattern of increased damage can be observed in P treated H&E stained sections. C: C57BL/10; U: untreated *mdx* mice, P: Prednisolone treated *mdx* mice, LS: Lisinopril-Spironolactone treated *mdx* mice; LSP, Lisinopril-Spironolactone-Prednisolone treated *mdx* mice; TS: Losartan-Spironolactone treated *mdx* mice; TSP: Losartan-Spironolactone-Prednisolone treated *mdx* mice. Bar = 100 µm.

Histopathological analysis was performed on the heart (Figure 5) and the quadriceps muscle (Figure 6). The quadriceps muscle was selected for this analysis because it is a large, mixed fiber limb muscle important for ambulation that is impacted by dystrophy in both mice and humans, and for comparison with the many published *mdx* studies using this muscle type. Hematoxylin and eosin (H&E) staining was used to observe the overall pathology of the muscle. Staining for mouse immunoglobulin G was used to detect damaged cardiac and skeletal muscle. Since muscle enzymes leak out of damaged muscle into serum and serve as the clinical diagnostic markers of muscle damage, likewise, serum proteins leak into the damaged tissue and allow quantitation of damage (Figures 5 and 6). Hearts from *mdx* untreated mice showed only minor cardiac pathology at 20 weeks-of-age (Figure 5), in contrast to the much more severe cardiac pathology in previous studies of 20 week het mice [15]. Only prednisolone treated mice consistently showed severe cardiac pathology by both IgG and H&E staining (Figure 5).

In quadriceps muscles, untreated, LS, and TS-treated *mdx* groups showed some myofiber damage and fibers with centrally-located nuclei, indicating previous degeneration and regeneration (Figure 6). Prednisolone showed severe overall pathology (Figure 6).

**Figure 7:**
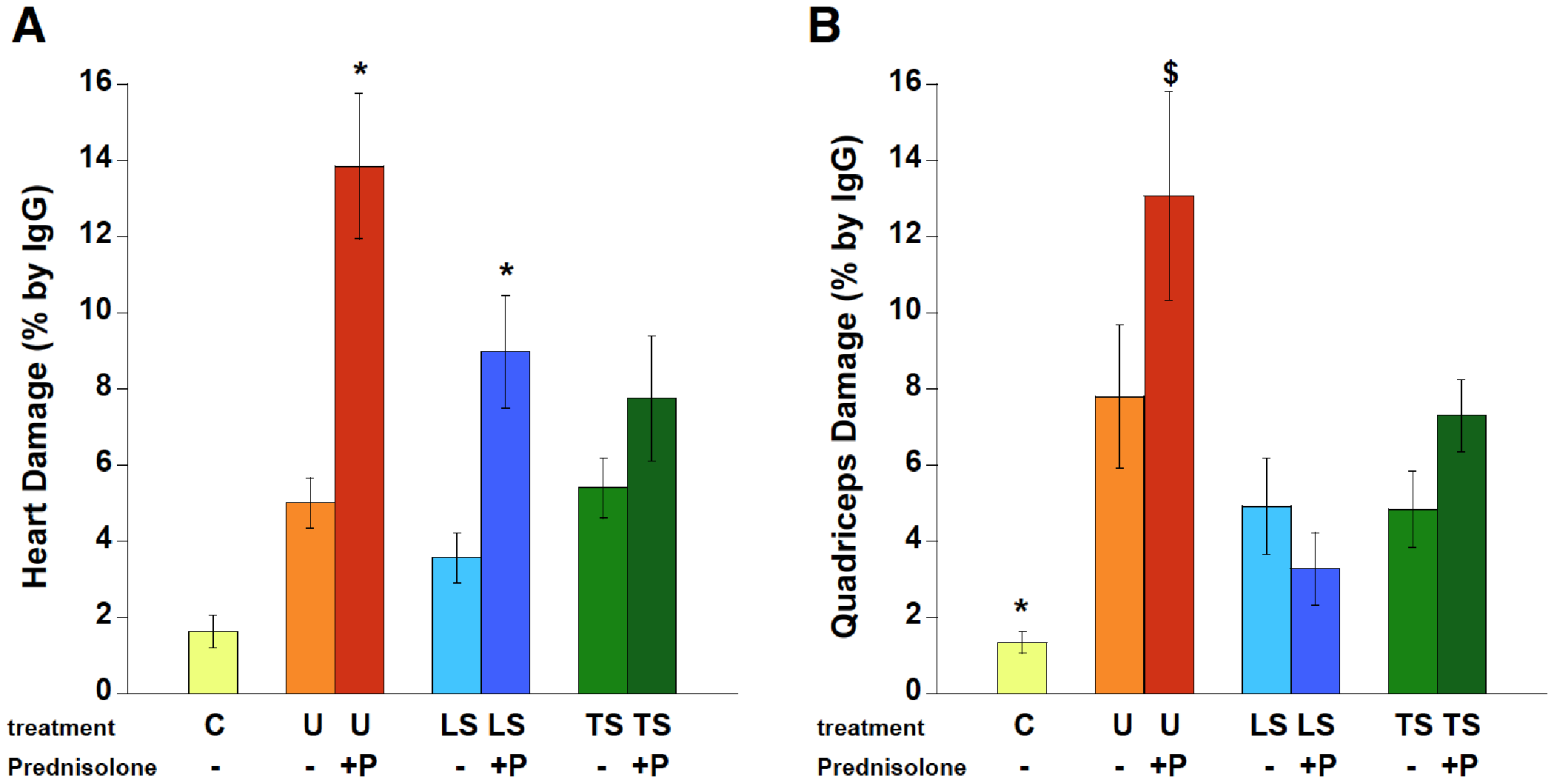
Quantification of histological analysis. A: Cardiac damage, quantified by the percentage of IgG staining, was significantly greater with Prednisolone inclusion in treatment (ANOVA). B: Quadriceps damage, quantified by % IgG staining, was significantly greater with Prednisolone in *mdx* mice, but had no significant impact in other groups. C57BL/10, n = 8; U: untreated *mdx* mice, n = 10, P: Prednisolone treated *mdx* mice, n = 9–10, LS: Lisinopril-Spironolactone treated *mdx* mice, n = 10; LSP, Lisinopril-Spironolactone-Prednisolone treated *mdx* mice n = 6; TS: Losartan-Spironolactone treated *mdx* mice, n = 10–11; TSP: Losartan-Spironolactone-Prednisolone treated *mdx* mice, n = 7–8. * indicates a significantly different value (P<0.05) compared to all other groups. ^$^ indicates a significantly higher value compared to the equivalent primary treatment without Prednisolone, P<0.05.

**Figure 8:**
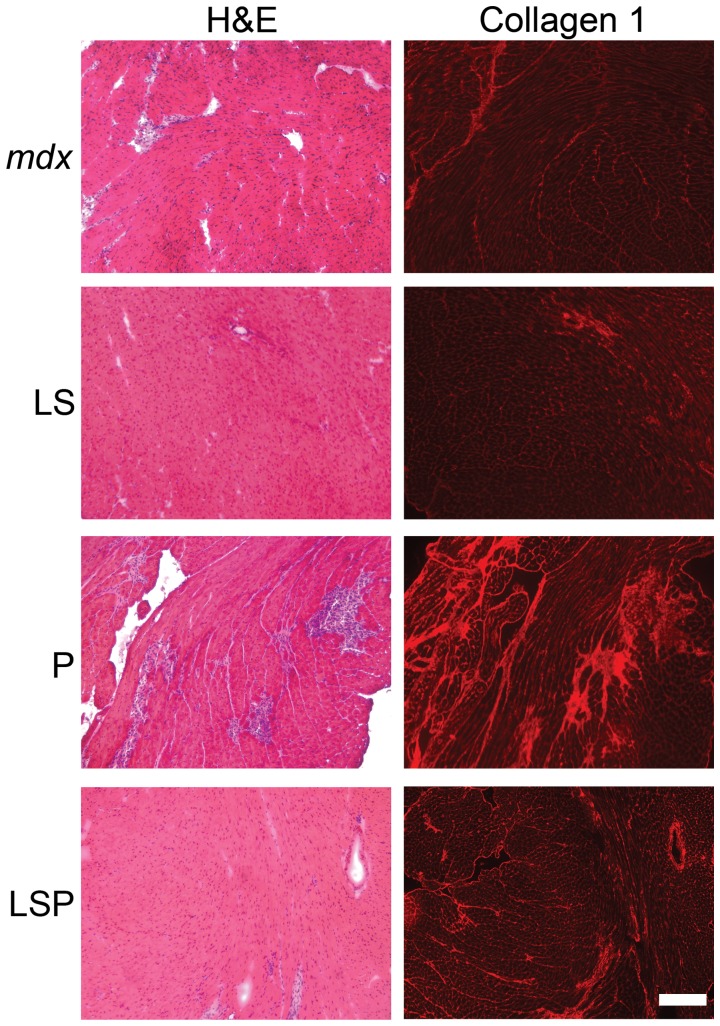
Collagen I immunostaining of hearts from Prednisolone treated mice confirms the presence of Collagen scarring replacing cardiac muscle. The damage present in hearts from P treated mice has progressed to collagen-containing fibrotic scarring in 20 week-old mice. LS shows no negative effects on *mdx* hearts. C: C57BL/10; U: untreated *mdx* mice, P: Prednisolone treated *mdx* mice, LS: Lisinopril-Spironolactone treated *mdx* mice; LSP, Lisinopril-Spironolactone-Prednisolone treated *mdx* mice; TS: Losartan-Spironolactone treated *mdx* mice; TSP: Losartan-Spironolactone-Prednisolone treated *mdx* mice. Bar = 100 µm.

To quantify the amount of damaged muscle, IgG staining was quantified as the percentage of total area in composited images covering most of quadriceps or left ventricle. In the heart, untreated *mdx* mice had 5.0±0.7% damaged area, versus only 1.6±0.4% in the C group (Figure 7A). ANOVA, followed by Dunnett, revealed P-treated animals had a significantly (P<0.05) higher percentage of damage (13.8±1.9%) than all other groups, and was significantly impacted by treatment (P = 0.00016 by ANOVA). (Figure 7B). In quadriceps muscle, damage was virtually absent in healthy control mice (C) (1.3±0.3%), and was markedly higher in untreated *mdx* mice (U) (7.8±1.9%). Most obvious was the very large amount of damage in the prednisolone treated animals (P) (13.1±2.7%), which was significantly (P<0.05) higher compared to C, LS, LSP, and TS groups. Further analysis showed that damaged regions also contained collagen scars in the hearts from P-treated mice (Figure 8), further emphasizing the negative impact of prednisolone.

Control mice had, as expected, only very few centrally located nuclei (1.4±0.5%). In all other groups, this number was significantly (P<0.0001) higher (U: 72.8±5.1%, P: 57.0±5.4%, LS: 72.1±2.7%, LSP: 65.6±1.2%, TS: 65.9±6.4%, and TSP: 46.0±5.5%). Between these *mdx* groups, only TSP was significantly different (P<0.05), and only vs. LSP and U mice.

## Discussion

Treatment with FDA approved drugs lisinopril and spironolactone showed a striking and significant improvement in both cardiac and skeletal muscle function and histopathology in the *utrn* ^+/−^;*mdx* mouse model of DMD [15]. In that model, mice lack dystrophin and are also haploinsufficent for its partially compensating homolog utrophin [16]. In this study, we used the *mdx* mouse model, which is the genotypic and most widely used animal model to study DMD. As such, the strength of this *mdx* model is that it is well defined and a wealth of data is collected in this model that allows for comparison between studies (reviewed in [23]). However, unlike the het model, the *mdx* model has only a relatively mild contractile dysfunction phenotype; at 20 weeks of age, the functional deficit between wild-type healthy control mice and *mdx* mice is significantly smaller for skeletal muscle function than in the het model. Moreover, although some parameters of cardiac muscle function are already affected in young *mdx* mice [31], others, such as peak strain rate (this study), are not yet statistically significant compared to wild-type mice. The *mdx* model also shows quantitatively less muscle damage and fibrosis than the het model [16]. Despite these limitations, we show here that in the *mdx* model, the combination of lisinopril and spironolactone generally improved the phenotype of contractile dysfunction and pathology. Several assessed parameters that are indicators of the pathological condition of the *mdx* mouse, when compared to healthy wild-type mice, such as IgG uptake, diaphragm force and specific EDL force, are no longer significantly different from wild-type mice when these *mdx* mice are treated with lisinopril/spironolactone. Although significance was lower in this model due to the less severe deficits observed in *mdx* mice compared to *utrn* ^+/−^;*mdx* mice, lisinopril/spironolactone treatment resulted in 80% of normal muscle force in both studies.

The goal of this study was to provide a comprehensive outcome assessment of treatment with lisinopril/spironolactone and directly compare this to current treatment regimens widely used in DMD patients, with a main focus on the impact of prednisolone. In contrast to lisinopril/spironolactone treatment, steroid treatment with the main active compound prednisolone worsened some of the assessed pathological parameters, in agreement with other recent reports [24], [25]. Compared to untreated *mdx* mice, treatment with prednisolone resulted in increased cardiac and skeletal muscle IgG uptake, and showed a lower cardiac strain rate, while skeletal muscle function was not significantly impacted. Moreover, we found that when comparing groups that only differed in the presence or absence of prednisolone (i.e. *mdx* vs. P, LS vs. LSP, and TS vs. TSP), a significant detrimental impact of prednisolone was observed in many assessed parameters, with the most profound impact on both the function and pathology of the heart. In addition, the triple treatments also resulted in increased mortality during the study, although dosages for this study were not optimized for this triple treatment approach and used previous efficacious dosages to optimize the comparison with previous studies. No clear trend towards improvement was observed in centrally located nuclei counts.

Although the impact of steroids on the heart is not consistent in all previous studies, our studies are in close agreement with recent reports showing a significant detrimental impact of steroids on the dystrophic heart [14], [24], and the present study now shows that this negative impact persists in the presence of drugs that generally improve these parameters. In humans, several studies have shown negative impact of long-term steroid use on non-dystrophic heart, including increased occurrence of atrial fibrillation [26] and increased water retention leading to higher blood pressure [27]. Interestingly however, prednisolone did prevent the small increase in body weight that is typically observed in *mdx* mice. In all direct comparison groups, addition of prednisolone to the treatment regimen led to a final body weight similar to control, whereas treatment with lisinopril/spironolactone and losartan/spironolactone significantly increased body weight. The same phenomenon was observed in the EDL muscle weight, all groups where prednisolone was part of the treatment regimen had smaller EDL muscles compared to the non-prednisolone groups. Body weight and EDL size were tightly linked, indicating the size of the EDL muscle was proportional to the body weight.

Losartan, in combination with spironolactone, showed effects that were most similar to those observed with lisinopril and spironolactone, with direct comparison of TS and LS revealing no significant differences. The only significant difference in our studies was that EDL specific force in TS treated *mdx* mice remained significantly lower than wild-type control mice, whereas LS-treated animals were not significantly lower than controls. Angiotensin II receptor antagonists are widely used clinically for treatment of heart failure; their main functional impact on the heart is through a reduction in afterload [28]. In addition, its positive impact on cardiac function in *mdx* mice [29], [30] make this drug relevant for DMD patients.

Limitations of the current study mainly stem from the logistical restrictions on the study design. In order to have a direct comparison between treatment groups that were handled and assessed in parallel, group sizes on n = 10–13 were maximal given the comprehensive analysis needed. The large number of different treatment groups were deemed necessary during the design of this project in collaboration with patient advocates, in order to provide a comprehensive comparison with current drug treatment regimens commonly used in the DMD patient population. Given these constraints, the mild phenotype of the *mdx* mice prevents a detailed quantitative comparison of individual parameters. Now that the same trend in the potential efficacy with lisinopril/spironolactone for dystrophic cardiac and skeletal muscles was replicated in this study with the genotypic *mdx* model, future studies would ideally be performed in more severely affected dystrophic mice as used by us and others in the past [15], [31], [32], [33]. Use of models with deficits in skeletal and cardiac function and pathology more similar to patients can be used to avoid the logistical and financial complications of the extremely large group sizes or lengthy study needed to tease out therapeutic effects in less affected *mdx* mice.

In order to optimize the potential clinical use of ACEi and aldosterone antagonists, which bind to mineralocorticoid receptors, future studies will require identification of the best drug combination and optimization of timing and dosage. More specific FDA approved mineralocorticoid receptor antagonists are available and can be tested for improved efficacy. In addition, testing different dosages of each drug alone and in combination will be required to determine whether these drugs can result in more than the 80% of normal muscle force observed in both studies.
